# High Diversity of *Planctomycetes* in Soils of Two Lichen-Dominated Sub-Arctic Ecosystems of Northwestern Siberia

**DOI:** 10.3389/fmicb.2016.02065

**Published:** 2016-12-22

**Authors:** Anastasia A. Ivanova, Irina S. Kulichevskaya, Alexander Y. Merkel, Stepan V. Toshchakov, Svetlana N. Dedysh

**Affiliations:** ^1^Winogradsky Institute of Microbiology, Research Center of Biotechnology of the Russian Academy of SciencesMoscow, Russia; ^2^Immanuel Kant Baltic Federal UniversityKaliningrad, Russia

**Keywords:** *Planctomycetes*, tundra wetland, lichen-dominated forested tundra, high-throughput 16S rRNA gene sequencing, FISH, cultivation studies

## Abstract

A wide variety of terrestrial ecosystems in tundra have a ground vegetation cover composed of reindeer lichens (genera *Cladonia* and *Cetraria*). The microbial communities of two lichen-dominated ecosystems typical of the sub-arctic zone of northwestern Siberia, that is a forested tundra soil and a shallow acidic peatland, were examined in our study. As revealed by molecular analyses, soil and peat layers just beneath the lichen cover were abundantly colonized by bacteria from the phylum *Planctomycetes*. Highest abundance of planctomycetes detected by fluorescence *in situ* hybridization was in the range 2.2–2.7 × 10^7^ cells per gram of wet weight. 16S rRNA gene fragments from the *Planctomycetes* comprised 8–13% of total 16S rRNA gene reads retrieved using Illumina pair-end sequencing from the soil and peat samples. Lichen-associated assemblages of planctomycetes displayed unexpectedly high diversity, with a total of 89,662 reads representing 1723 operational taxonomic units determined at 97% sequence identity. The soil of forested tundra was dominated by uncultivated members of the family Planctomycetaceae (53–71% of total *Planctomycetes*-like reads), while sequences affiliated with the *Phycisphaera*-related group WD2101 (recently assigned to the order Tepidisphaerales) were most abundant in peat (28–51% of total reads). Representatives of the *Isosphaera*–*Singulisphaera* group (14–28% of total reads) and the lineages defined by the genera *Gemmata* (1–4%) and *Planctopirus–Rubinisphaera* (1–3%) were present in both habitats. Two strains of *Singulisphaera*-like bacteria were isolated from studied soil and peat samples. These planctomycetes displayed good tolerance of low temperatures (4–15°C) and were capable of growth on a number of polysaccharides, including lichenan, a characteristic component of lichen-derived phytomass.

## Introduction

Members of the bacterial phylum *Planctomycetes* inhabit a wide range of aquatic and terrestrial environments with diverse environmental conditions. Yet, most cultured and taxonomically characterized representatives of this phylum are mesophiles (Ward, 2010). Several moderately thermophilic planctomycetes were also described (Giovannoni et al., 1987; Kovaleva et al., 2015; Slobodkina et al., 2015) but psychrophilic members of this phylum are not yet known. At the same time, planctomycetes are commonly detected in various low-temperature ecosystems by molecular surveys. In permafrost-affected soils of arctic and sub-arctic tundra, they comprise one of the minor groups (several percent of total diversity) of the bacterial community (Steven et al., 2007; Wagner et al., 2009; Kim et al., 2014; Hultman et al., 2015). In some habitats, however, planctomycetes may be significantly more abundant. For example, they comprise up to 20% of total bacterial diversity in biological soil crusts inhabiting polar desert soils at the northern land limit of the arctic polar region (Steven et al., 2013).

*Sphagnum*-dominated boreal wetlands represent one of the most extensive terrestrial low-temperature environments where members of the *Planctomycetes* are especially widespread and abundant (Dedysh et al., 2006; Kulichevskaya et al., 2006; Bragina et al., 2012, 2015; Ivanova and Dedysh, 2012; Serkebaeva et al., 2013; Moore et al., 2015; Ivanova et al., 2016). The predominant planctomycete populations in northern wetlands are represented by members of the phylogenetic group defined by the genera *Isosphaera* and *Singulisphaera* (Ivanova and Dedysh, 2012; Serkebaeva et al., 2013; Moore et al., 2015). This group was recently given the status of a separate family, i.e., the family Isosphaeraceae (Kulichevskaya et al., 2016). Several taxonomically characterized representatives of this family from northern peatlands, such as *Singulisphaera acidiphila*, *Singulisphaera rosea*, and *Paludisphaera borealis*, are psychrotolerant bacteria that are capable of growth at low temperatures, down to 4–6°C (Kulichevskaya et al., 2008, 2012a, 2016). Most currently described peat-inhabiting planctomycetes have the ability to degrade various heteropolysaccharides and appear to be involved in degradation of *Sphagnum*-derived litter (Kulichevskaya et al., 2007; Moore et al., 2015). The proportion of *Sphagnum*-dominated wetlands declines in high-latitude regions, where mosses became replaced with lichens. Lichens cover approximately 6–8% of the Earth’s land surface (Haas and Purvis, 2006; Asplund and Wardle, 2016). In some forests, drylands and tundras they can make up most of the ground layer biomass (Asplund and Wardle, 2016). For example, in Yamal peninsular, lichens cover up to 40% of the whole vegetated tundra area (Tsibulsky, 1995). Although the component composition of lichen thalli received significant research attention (Cardinale et al., 2008; Grube et al., 2009; Bates et al., 2011; Hodkinson et al., 2012; Zhang et al., 2015), microbial communities of lichen-dominated tundra soils remain poorly studied. We hypothesized that, similar to *Sphagnum*-dominated boreal wetlands, planctomycetes may also be abundant in lichen-covered wetlands and upland soils of tundra. To verify this hypothesis, the present study was initiated by focusing on two distinct ecosystems within the zone of forested tundra and discontinuous permafrost in northwest Siberia with a ground vegetation cover composed of reindeer lichens (genera *Cladonia* and *Cetraria*).

## Materials and Methods

### Sampling Sites

**FIGURE 1:**
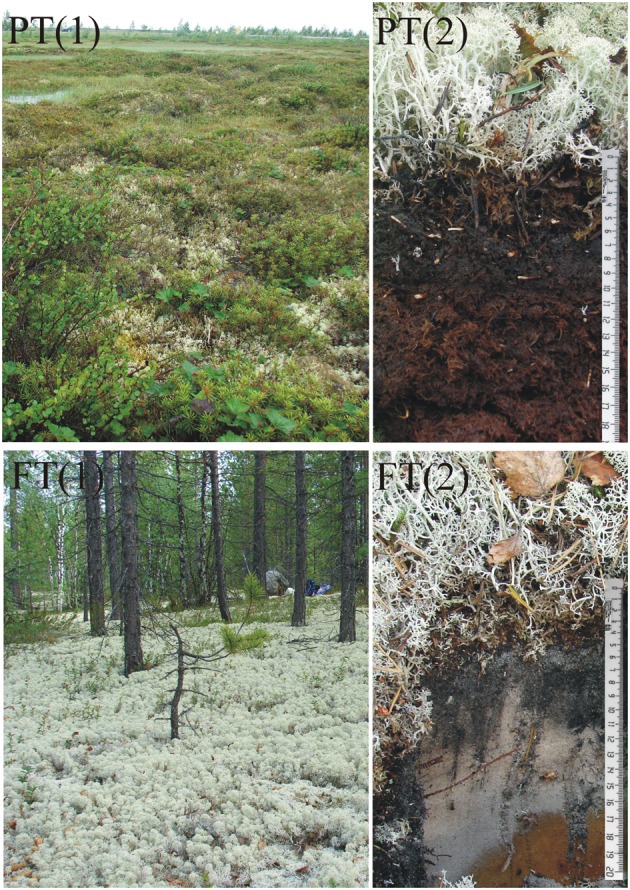
**Two different tundra sites examined in this study: peatland site [PT(1)] with mosaic cover of lichens and mosses and pine forest [FT(1)] with ground vegetation cover composed of reindeer lichens.** Depth profiles of peat and forest soils [PT(2), FT(2)].

This study was performed in the Nadym region of northwest Siberia, Yamalo-Nenets Autonomous Okrug, Russia, within the zone of forested tundra and discontinuous permafrost (Matyshak et al., in press). The climate is mid-continental with very cold winters. According to the Nadym meteorological station records, mean annual air temperature is 5.9°C (10.8°C in summer and -14.2°C in winter). Mean annual ground temperature fluctuates between +1 and -3°C (Pavlov and Moskalenko, 2002). Two contrasting study sites differing by the degree of hydromorphism were selected, that is a shallow peatland (N65°35′01.3″, E73°03′10″) and lichen-dominated forested tundra (N65°36′07.1″, E72°44′39.5″). The distance between these study sites was about 14.5 km. A mosaic vegetation cover of the studied peatland was composed of lichens and mosses including *Cladonia stellaris*, *Cladonia sylvatica*, and *Sphagnum* spp. [**Figure 1**, PT(1)]. *Betula nana*, *Rubus chamaemorus*, *Ledum* sp., *Vaccinium uliginosum*, *Vaccinium myrtillus*, *Eriophorum* sp., and *Carex* sp. were also present. The peatland profile included a substantial fraction of live non-vascular vegetation and a poorly decomposed acidic (pH 4.4) peat horizon [**Figure 1**, PT(2)]. During the sampling time in July 2014, permafrost table was a depth of 25–30 cm. The second study site was located in a pine (*Pinus sibirica*) forest within a permafrost-free zone. The ground vegetation was composed of lichens (*C. stellaris*, *Cladonia alpestris*, and *Cetraria islandica*) with a minor presence of *Vaccinium* spp., *Ledum palustre*, and *Polytrichum commune* [**Figure 1**, FT(1)]. The soil profile included litter (depth 0–1 cm), thin organic layer (1–8 cm), gray sand (8–16 cm), and sandy subsoil [**Figure 1**, FT(2)]. The surface organic layer had a pH 4.1, while sandy subsoil was nearly neutral (pH 6.1). Three individual plots, on a distance of approximately 20–30 m from each other, were chosen within each study site for sampling purposes. The peat and soil samples (each sample of approximately 500 g) were collected over the profiles of these six experimental plots. The samples were transported to the laboratory in boxes containing ice packs, homogenized, and fixed for fluorescent *in situ* hybridization (FISH) or frozen at -20°C for DNA extraction within 1 day after sampling.

### Fluorescent *In situ* Hybridization

The soil and peat samples were fixed with 4% (wt/vol) freshly prepared paraformaldehyde solution as described by Dedysh et al. (2001) and Ivanova and Dedysh (2012). A combination of two Cy3-labeled oligonucleotide probes PLA46 (5′-GACTTGCATGCCTAATCC-3′) and PLA886 (5′-GCCTTGCGACCATACTCCC-3′) (Neef et al., 1998), which was used in our previous studies on *Sphagnum*-dominated boreal wetlands, was applied for specific detection of planctomycetes. One additional, *Planctomycetes*-specific probe PLA929 (5′-CCACCGCTTGTGTGAGC-3′) was designed in this study based on SILVA database (Quast et al., 2013) and PRIMROSE software (Ashelford et al., 2002). All oligonucleotide probes were purchased from Syntol (Moscow, Russia). Hybridization was done on gelatin-coated (0.1%, wt/vol) and dried Teflon-laminated slides (MAGV, Germany) with eight wells for independent positioning of the samples. The fixed samples were applied to these wells, hybridized to the corresponding fluorescent probes, and stained with the universal DNA stain 4′,6-diamidino-2-phenylindole (DAPI, 1 μM) as described earlier (Dedysh et al., 2001). The cell counts were carried out with a Zeiss Axioplan 2 microscope (Zeiss, Jena, Germany) equipped with the Zeiss Filters No 20 and 02 for Cy3-labeled probes and DAPI staining, respectively. Cell counting was performed on 100 randomly chosen fields of view (FOV) for each test sample. The number of target cells per gram of wet peat was determined from the area of the sample spot, the FOV area, the volume of the fixed sample used for hybridization, and the volume of the peat water extracted from the sample.

### High-Throughput Sequencing of 16S rRNA Genes

Extracts of total DNA used for molecular diversity studies were obtained from the samples collected from the surface (2–5 cm depth) organic soil layers underlying lichen cover. Six individual samples (three samples from the forested tundra site and three samples from the peatland site), each of 0.5 g wet weight, were taken for the analysis and processed separately. Isolation of total DNA from peat samples was performed using FastDNA SPIN kit for soil (MP Biomedicals, USA) and FastPrep-24 homogenizer (MP Biomedicals, USA) in accordance with manufacturer’s instructions. Subsequent purification of DNA samples was performed using preparative gel electrophoresis and Cleanup Standard Kit (Evrogen, Russia). The V4 region of 16S rRNA genes was amplified from the DNA samples using the 515f/806r primer set (Caporaso et al., 2011) with some primer modifications: as forward primer the Univ515F primer was used (5′-GTG BCA GCM GCC GCG GTA A-3′; Kublanov et al., 2009). The resulting amplicons were purified by agarose gel electrophoresis using Cleanup Standard Kit (Evrogen, Russia) and sequenced using the 300PE protocol on MiSeq System (Illumina, USA). Resulting reads were subjected to stringent quality filtering and trimming with CLC Genomics Workbench 7.5 (Qiagen, Germany). After filtering, overlapping paired-end library reads were merged with SeqPrep tool (https://github.com/jstjohn/SeqPrep). Demultiplexing and further processing of the resulting data set was carried out using QIIME v.1.8. package (Caporaso et al., 2010). Taxonomy assignment was performed using Ribosomal Database Project (RDP) classifier retrained with Silva 119 database (Pruesse et al., 2007; Quast et al., 2013). Culling of chimeric sequences was performed using ChimeraSlayer algorithm (Grabherr et al., 2011).

### Statistical Analyses

Statistical evaluations were made with GraphPad Prism (v. 7.0) applying multiple *t*-tests with false discovery rate (FDR) approach (desired *Q* = 1%). Two-stage step-up method of Benjamini, Krieger, and Yekutieli (Benjamini et al., 2006) was used to control the FDR.

### Cultivation Studies

The enrichment strategy, which gives a selective advantage to planctomycetes, and the isolation approach have been described elsewhere (Kulichevskaya et al., 2012b). Briefly, 2 g of wet peat were suspended in 10 ml of sterile water and treated in a laboratory stomacher at 240 rpm for 5 min. The resulting peat suspension was used to inoculate 500-ml serum bottles containing 90 ml of sterile dilute mineral medium M1 of the following composition (gram per liter of distilled water): KH_2_PO_4_, 0.1; (NH_4_)_2_SO_4_, 0.1; MgSO_4_ × 7H_2_O, 0.1; CaCl_2_ × 2H_2_O, 0.02; 1 ml of trace element solution “44” and 1 ml Staley’s vitamin solution (Staley et al., 1992); pH 4.8–5.5. The bottles were tightly closed and incubated in the dark at 15°C. After 4 weeks of incubation, 20 μl aliquots of the resulting enrichment cultures were spread plated onto medium M31 (modification of medium 31 described by Staley et al., 1992), solidified with 10 g phytagel (Sigma-Aldrich), containing (per liter distilled water): 0.1 g KH_2_PO_4_, 20 ml Hutner’s basal salts, 1.0 g *N*-acetylglucosamine, 0.2 g ampicillin (sodium salt), 0.1 g peptone, 0.1 g yeast extract, pH 5.8. The plates were then incubated at 22°C for 4 weeks. Colonies and microbial cell masses that developed on plates were screened microscopically for the presence of budding cells with planctomycete-like morphology. The selected cell material was re-streaked onto the same medium M31, supplemented with 0.05% glucose. The resulting isolates were identified by means of comparative 16S rRNA gene sequence analysis. PCR-mediated amplification of the 16S rRNA gene from positions 28 to 1491 (numbering according to the International Union of Biochemistry and Molecular Biology nomenclature for *Escherichia coli* 16S rRNA) was performed using primers 9f and 1492r and reaction conditions described by Weisburg et al. (1991). The 16S rRNA gene amplicons were purified using QIAquick spin columns (Qiagen) and sequenced on an ABI Prism 377 DNA sequencer using BigDye terminator chemistry, as specified by the manufacturer (PE Applied Biosystems). Phylogenetic analysis was carried out using the ARB program package (Ludwig et al., 2004).

Physiological tests were performed in liquid medium M31. Growth of novel isolates was monitored by nephelometry at 600 nm in a BioPhotometer (Eppendorf, Germany). The capability to degrade different biopolymers was examined by measuring the rate of CO_2_ production in tightly closed 160 ml serum bottles containing 10 ml of liquid medium M1 with 0.005% yeast extract as a growth factor and 0.05% (w/v) of the corresponding polymer substrate for 1 month at 22°C. Control incubations were run in parallel under the same conditions but without a polymer substrate. CO_2_ concentration was measured with a non-dispersive infra-red gas-analyzer “Infralit” (Germany). All experiments were performed in triplicate.

### Sequence Accession Numbers

The 16S rRNA gene reads retrieved using Illumina pair-end sequencing from the soil and peat samples (raw data) have been deposited under the Bioproject number PRJNA344855 in the NCBI Sequence Read Archive, with the accession numbers SAMN05846487 and SAMN05846486 for the peatland and forested tundra soil, respectively. The GenBank/EMBL/DDBJ accession number for the 16S rRNA gene sequence of strain P12 is KX943553.

## Results

### Detection of *Planctomycetes* in Tundra Ecosystems by FISH

**FIGURE 2:**
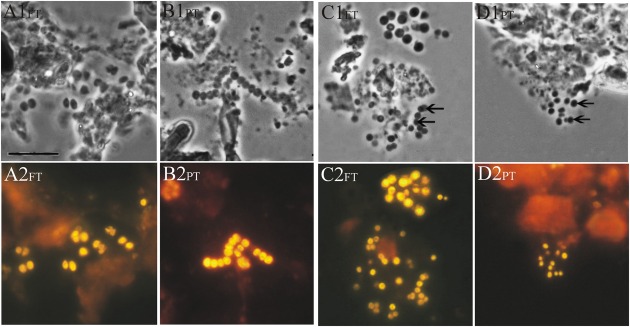
**Specific detection of planctomycete cells in a forested tundra soil (FT) and peat (PT) samples by FISH.** Phase-contrast images **(A1–D1)** and the respective epifluorescent micrographs of *in situ* hybridizations with Cy3-labeled probes **(A2–D2)** are shown. Hybridizations with the probes PLA46 + PLA886 are displayed in panels **(A2)** and **(B2)**, while hybridizations with the probe PLA929 are shown in panels **(C2)** and **(D2)**. Bars, 10 μm.

The preliminary screening for the presence of planctomycetes in lichen-dominated forested tundra and a discontinuous permafrost peatland was performed by FISH using a combination of two Planctomycetes-specific oligonucleotide probes PLA46 and PLA886, which were applied in our previous studies of *Sphagnum*-dominated boreal wetlands (Kulichevskaya et al., 2006; Ivanova and Dedysh, 2012). These probes hybridized to relatively large (2–3 μm) spherical or ovoid-shaped cells that were arranged in chains or in shapeless cell aggregates (**Figures 2A,B**) and morphologically resembled those commonly observed in *Sphagnum*-derived peat. Since the target specificity of the probes PLA46 and PLA886 is restricted by the orders Planctomycetales and Candidatus Brocadiales and does not cover all currently known diversity within the phylum *Planctomycetes*, one additional probe, PLA929, was designed and applied in our study. This novel probe displayed good diversity coverage for the order Planctomycetales and, in contrast to PLA46 and PLA886, was also specific for members of the order Phycisphaerales as well as for several uncultivated phylogenetic sub-groups of planctomycetes (detection spectra of these probes are compared in Supplementary Figure S1). Notably, the cells detected in our samples by the probe PLA929 were morphologically more diverse than those revealed by the combination of PLA46 and PLA886. In addition to large spherical or ovoid-shaped cells detected by the conventional probe set, the probe PLA929 hybridized also to smaller (0.8–1.5 μm) cells assembled in round-shaped conglomerates (**Figures 2C,D**). The occurrence of well-developed slimy capsules around the cells detected by the probes was highly typical for the samples from tundra.

**FIGURE 3:**
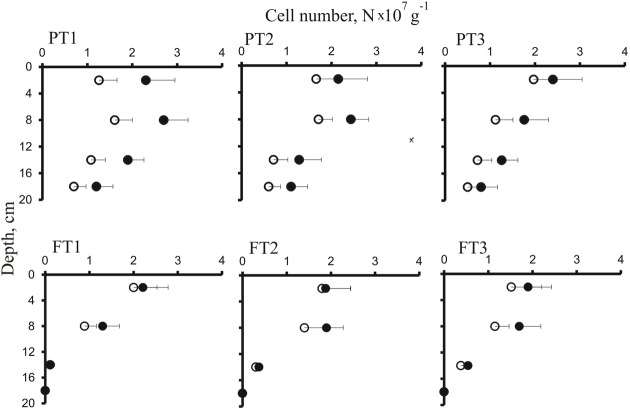
**Depth distribution of cells targeted by FISH with the probes PLA46 + PLA886 (open circles) and the probes PLA46 + PLA886 + PLA929 (closed circles) in soil sampled from the peatland (PT1–PT3) and forested tundra (FT1–FT3).** Values are means from 100 randomly chosen counting fields of view; error bars indicate standard errors.

The abundance and distribution of planctomycetes within the depth profiles of a forested tundra soil and a shallow peatland were further examined using two equimolar mixtures of probes, the conventional set PLA46 + PLA886 and the improved set PLA46 + PLA886 + PLA929 (**Figure 3**). An apparent increase in detection efficiency due to the use of PLA929 was most evident in samples from the peatland suggesting the presence of *Phycisphaera*-like planctomycetes. In both ecosystems, highest abundances of cells targeted with *Planctomycetes*-specific probes (1.3–2.3 × 10^7^ cells per gram of wet soil or peat) were observed in the surface organic layers. Depth distribution patterns of planctomycetes in a forested tundra soil and a peatland, however, were somewhat different. In a forested tundra soil, these bacteria were localized in a relatively thin (6–8 cm) surface organic layer, just beneath the lichen cover, and sharply declined in abundance in subsurface sandy soil. By contrast, they were more evenly distributed over the profile of a shallow peatland and were present in all peat layers above the permafrost. The soil and peat samples from the uppermost organic layers, which were most abundantly colonized by planctomycetes (depth 2–5 cm), were used for the high-throughput molecular diversity analysis.

### Sequencing Statistics and Microbial Community Composition

A total of 1,278,169 partial 16S rRNA gene sequences (mean amplicon length 253 bp) were retrieved from the examined soil and peat samples. Of these, 1,071,404 reads were retained after quality filtering and denoising of the raw data. Only a minor part of these sequences (0.1–0.4%) were classified as belonging to the Archaea, more specifically to the uncultivated members of the Thaumarchaeota. Major groups of bacteria in the study sites were represented by the Acidobacteria (22–24 and 31–40% of all classified 16S rRNA gene sequences in a forested tundra and a shallow peatland, respectively), Proteobacteria (33–40 and 34–35%), *Planctomycetes* (13–16 and 8–10%), Actinobacteria (11–13 and 6–14%), and Verrucomicrobia (9–10 and 5–9%) (Supplementary Figure S2). Minor groups (relative abundance ≤2% of all classified sequences) included Chloroflexi, Armatimonadetes, Bacteroidetes, Chlamydiae, Firmicutes, Cyanobacteria, Elusimicrobia, Gemmatimonadetes and the candidate divisions TM6 and WD272. Statistically significant differences between the relative abundances of particular bacterial groups in a forested tundra soil and a peatland were detected only for the Acidobacteria and *Planctomycetes* (Supplementary Table S2).

### High-Throughput Analysis of Planctomycete Diversity

The pool of 16S rRNA gene fragments from the *Planctomycetes* obtained in our study included 127,959 reads. Nearly equal numbers of reads (∼12,000–16,000) were obtained from the three samples from a peatland (PT1–PT3) and two samples from a forested soil (FT1 and FT3). The pool of reads retrieved from the third sample of a forested soil (FT2) was much larger than the others (54,135 reads) and, therefore, only ∼16,000 reads were randomly selected from this sequence pool for further analysis (**Table 1**). The final set of 16S rRNA gene fragments from the *Planctomycetes* used for the analysis included 89,662 reads.

**Table 1:** Sequencing statistics and various alpha-diversity metrics.

Ecosystem	Sample ID	Raw reads	Filtered reads^∗^	Planctomycete reads	Diversity indices				
					Chao1	Shannon	Observed species	Menhinick	Good’s coverage estimator
Forested tundra	FT1	128,572	104,964	14,852	1,165	7.37	842	6.91	0.98
	FT2	402,892	339,722	54,135 (15,838)	1,828 (1,108)	7.56 (7.42)	1,641 (875)	7.05 (6.95)	0.99 (0.98)
	FT3	151,288	125,631	16,191	1,252	7.31	932	7.32	0.98
Tundra peatland	PT1	133,623	114,080	16,209	847	6.12	610	4.79	0.99
	PT2	220,555	186,108	14,712	932	6.93	677	5.58	0.98
	PT3	241,239	200,899	11,860	860	6.79	619	5.68	0.98

*^∗^Filtered reads: number of sequences excluding singletons and chimera sequences. The randomized number of sequences is given in parentheses for the overrepresented sample FT2.*

As evidenced by Good’s coverage estimator, our sequencing effort covered 98–99% of the total planctomycete diversity in examined samples (**Table 1**). The assemblages of planctomycetes were more diverse in a forested tundra soil (Chao1 1108–1252 and Shannon index 7.31–7.42) than in a peatland (Chao1 847–932 and Shannon index 6.12–6.93). The number of species-level operational taxonomic units (OTUs) determined at 97% sequence identity ranged between 842 and 932 in a forested soil and between 610 and 677 in a peatland. Beta-diversity analysis revealed that the assemblages of planctomycetes in the three samples from a forested tundra soil were similar to each other, but distinct to those in the three samples from a peatland (Supplementary Figure S3).

**FIGURE 4:**
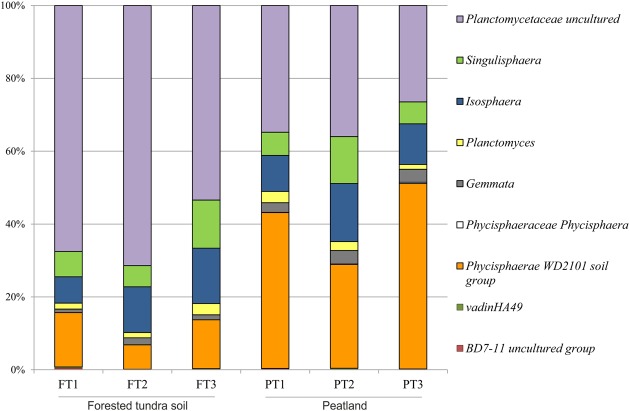
**Community composition of the *Planctomycetes* in three peatland sites (PT) and three sites of the forested tundra (FT) based on Illumina paired-end sequencing of 16S rRNA genes.** The results of statistical analysis of differences between relative abundances of particular groups of planctomycetes in two study sites are given in Supplementary Table S3.

Further taxonomic analysis revealed that soil samples from a forested tundra were dominated by uncultivated members of the family Planctomycetaceae (53–71% of total *Planctomycetes*-like reads), while sequences affiliated with the *Phycisphaera*-related group WD2101 were most abundant in peat (28–51% of total reads) (**Figure 4**). Representatives of the *Isosphaera*–*Singulisphaera* group (14–28% of total reads) and the lineages defined by the genera *Gemmata* (1–4%) and *Planctomycetes* (1–3%) were present in both habitats. As revealed by the statistical analysis (Supplementary Table S3), significant differences in relative abundances of particular planctomycete clades were observed only for the *Phycisphaera*-related group WD2101 and the group of uncultivated members of the family Planctomycetaceae.

**FIGURE 5:**
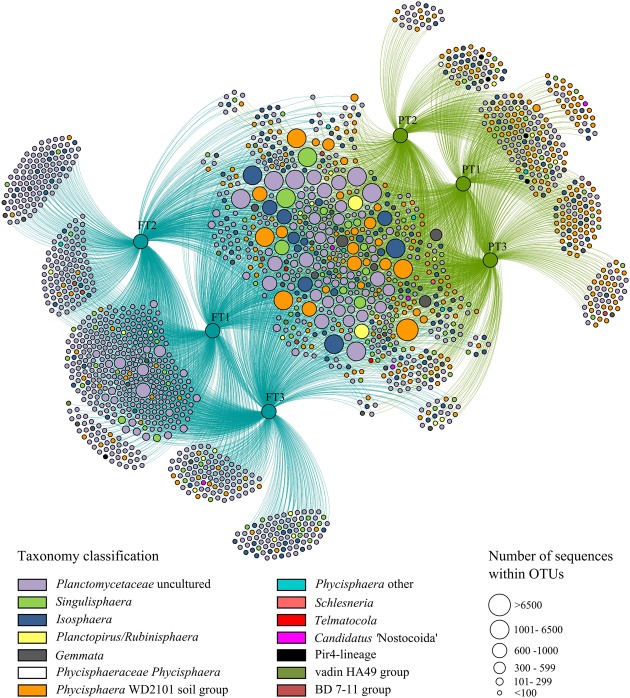
**Network graph of three peatland sites (green circles marked as “PT”) and three sites of forest tundra (blue circles marked as “FT”) based on occurrence of planctomycete operational taxonomic units (OTUs).** The colored nodes represent all OTUs that were identified in this study. The colors indicate the taxonomic assignment of each OTU and match those in **Figure 3**. The distance between sample nodes reflects their OTU connectivity; e.g., samples that share many OTUs are plotted close to each other, whereas those that share less OTUs are more distant to each other. The size of the OTU nodes is weighted according to the number of sequences comprising in the particular OTU from larger nodes with more sequences to smaller ones with less number of sequences.

**FIGURE 6:**
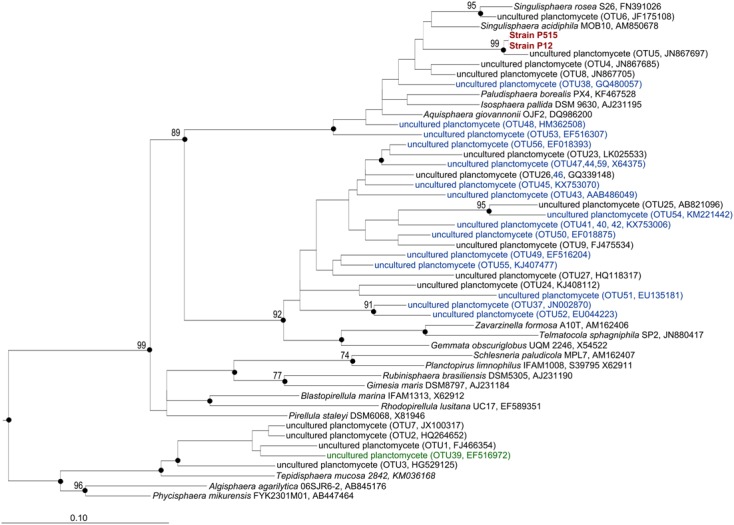
**16S rRNA gene-based neighbor-joining tree (Jukes–Cantor correction) showing the phylogenetic relationship of strains P12 and P515, OTUs from **Table 2** and Supplementary Table S1 to representative members of the *Planctomycetes*.** Unique OTUs identified only in the forested soil are indicated with blue color. OTUs that were found only in the peatland are indicated with green color. Black circles indicate that the corresponding nodes were also recovered in the maximum-likelihood and maximum-parsimony trees. The root (not shown) is composed of five 16S rRNA gene sequences from anammox planctomycetes (AF375994, AF375995, AY254883, AY257181, AY254882). Bar, 0.1 substitutions per nucleotide position.

In total, 1723 OTUs determined at 97% sequence identity were identified in our study for tundra-inhabiting planctomycetes. Of these, 342 OTUs were present in both ecosystems (148 OTUs were detected in all examined samples, 78 OTUs were detected in five samples and 116 OTUs were detected in four samples) (**Figure 5**). The OTUs comprising ≥2% of all *Planctomycetes*-affiliated reads in examined samples are listed in **Table 2** and are displayed in the phylogenetic tree in **Figure 6**. The most abundant OTUs were represented by uncultivated members of the *Phycisphaera*-related group WD2101 (OTUs No 1, 2, 3, 7) and uncultivated members of the family Planctomycetaceae (OTUs No 9, 23–27). *Singulisphaera*- (OTUs No 5 and 6), and *Isosphaera*-related planctomycetes (OTUs 4 and 8) were also well represented both in a forested soil and in a peatland (**Table 2**; **Figures 5** and **6**). Members of the genera *Gemmata*, *Planctopirus*, and *Rubinisphaera* were detected in both ecosystems but were less abundant (**Figure 6**).

**Table 2:** The most abundant operational taxonomic units (OTUs) and their relative abundance in a forested tundra soil (FT) and a shallow tundra peatland (PT).

OTU ID	Relative abundance of OTUs (%)		Taxonomy	Close GenBank match	Reported habitat	Similarity (%)
	PT	FT				
OTU1	12.1	2.9	Phycisphaerae WD2101 soil group	FJ466354	Volcanic deposit, Hawaii, USA	99
OTU2	5.6	0.9	Phycisphaerae WD2101 soil group	HQ264652	Forest soil, Taiwan	100
OTU3	4.2	1.6	Phycisphaerae WD2101 soil group	HG529125	Sphagnum moss, Finland	95
OTU4	2.4	0.8	*Isosphaera*	JN867685	Peat bog, Yaroslavl, Russia	99
OTU5	2.4	1.1	*Singulisphaera*	JN867697	Peat bog, Yaroslavl, Russia	99
OTU6	2.3	3.0	*Singulisphaera*	JF175108	Contaminated soil, China	100
OTU7	2.1	0.1	Phycisphaerae WD2101 soil group	JX100317	Coastal forest, Taiwan	98
OTU8	2.1	3.8	*Isosphaera*	JN867705	Peat bog, Yaroslavl, Russia	100
OTU9	2.0	0.4	Planctomycetaceae uncultured	FJ475534	Pine forest soil, Sweden	98
OTU23	0.0	4.3	Planctomycetaceae uncultured	LK025533	Peat soil, Germany	97
OTU24	0.4	3.9	Planctomycetaceae uncultured	KJ408112	Forest soil, Taiwan	94
OTU25	0.6	2.9	Planctomycetaceae uncultured	AB821096	Forest soil, Jeju, South Korea	98
OTU26	0.2	2.7	Planctomycetaceae uncultured	GQ339148	Freshwater seep, Denmark	96
OTU27	0.0	2.0	Planctomycetaceae uncultured	HQ118317	Eucalyptus forest, California, USA	99

*OTUs are defined at a 97% sequence identity threshold. Percentage values are calculated by relating the number of sequences recovered for the particular OTU to the total number of sequences derived from forested tundra soil or tundra peatland, respectively. OTUs with the relative abundance of >1% at least for one ecosystem are shown.*

Some OTUs, however, were present only in one of the two examined ecosystems (**Figure 5**). Most abundant (comprising ≥100 reads) of these unique OTUs are listed in Supplementary Table S1 and are shown in **Figure 6** (those present only in a peatland are shown in green, while those detected only in a forested soil are displayed in blue). In total, 749 unique OTUs were identified in a forested soil (208 OTUs were detected in three examined samples, 288 OTUs were detected in two samples and 240 OTUs were detected in one sample). The most abundant OTUs revealed in all three examined sites of a forested tundra were represented by uncultivated members of the family Planctomycetaceae (OTUs No 37, 38, 40, and 41) as well as by *Singulisphaera* species (OTUs No 48 and 53) (Supplementary Table S1; **Figure 6**). OTUs detected only in a single sample were more diverse and included uncultivated members of the family Planctomycetaceae and *Phycisphaera*-related group WD2101 as well as representatives of the genera *Isosphaera*, *Gemmata*, *Planctopirus*, and *Rubinisphaera* (**Figure 5**). The number of unique OTUs detected exclusively in a tundra peatland was 357 (63 OTUs were detected in three examined samples, 184 OTUs were detected in two samples and 108 OTUs were detected in one sample). The most abundant OTUs of planctomycetes specific for the peatland were represented by uncultivated members of the *Phycisphaera*-related group WD2101 (OTU 39, Supplementary Table S1; **Figure 6**).

### Characteristics of Isolated *Planctomycetes*

**FIGURE 7:**
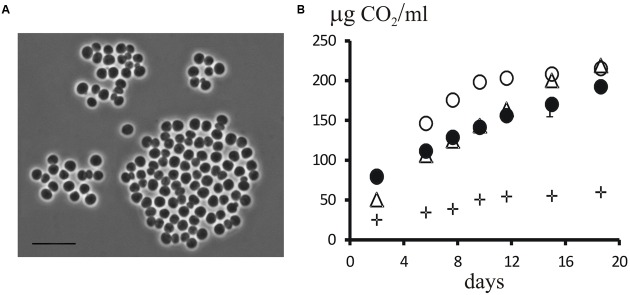
**(A)** Phase-contrast micrograph of cells of strain P12. Bar, 10 μm. **(B)** Dynamics of CO_2_ accumulation in the headspace of flasks during cultivation of strain P12 with sucrose (triangles), xylan (open circles), and lichenan (closed circles) as growth substrates. The control without added C-substrate is shown by crosses.

Two isolates of planctomycetes, strains P12 and P515, were obtained from a peatland and a forested tundra soil, respectively. These isolates were represented by large (2–3 μm) spherical, non-motile cells that multiplied by budding and occurred singly or were arranged in aggregates (**Figure 7A**). 16S rRNA gene sequences from strains P12 and P515 were identical and affiliated with the family Isosphaeraceae (Kulichevskaya et al., 2016). They displayed 93–94% sequence similarity to 16S rRNA gene sequences from members of the genus *Singulisphaera*, 91–92% sequence similarity to members of the genera *Paludisphaera*, and 89% sequence similarity to *Isosphaera pallida* (**Figure 6**). In general, phenotypic properties of strains P12 and P515 were similar to those defined for members of the genus *Singulisphaera* (Kulichevskaya et al., 2012a). Most likely, these isolates represent a novel species of this genus although their exact taxonomic identification requires obtaining additional chemotaxonomic and genotypic data. Strains P12 and P515 grew well on several polysaccharides, including starch, xanthan gum, and esculin. Notably, the growth rates on xylan and lichenan were comparable to those on sucrose (**Figure 7B**). Lichenan (or lichenin) (C_6_H_12_0_6_)_n_, is a cold-water insoluble, gel-forming, linear (1 → 3)-(1 → 4)-β-D-glucan occurring in certain species of lichens, including reindeer lichens (Huneck and Yoshimura, 1996; Olafsdottir and Ingólfsdottir, 2001). Good growth on this polysaccharide suggests involvement of strains P12 and P515 in degradation of lichen-derived debris in tundra ecosystems. The specific growth rate displayed by these planctomycetes at 10°C (μ = 0.014 h^-1^; T_d_ = 49.8 h) was close to that observed at 22°C (μ = 0.020 h^-1^; T_d_ = 35.3 h). Slow but consistent growth was also detected at 4°C (μ = 0.008 h^-1^; T_d_ = 82 h). In contrast to the previously described species of the genus *Singulisphaera*, strains P12 and P515 did not grow above 28°C. These planctomycetes, therefore, were psychrotolerant bacteria capable to develop within the low temperature range (4–15°C) characteristic for tundra ecosystems during summer season.

## Discussion

As revealed in our study, soil and peat layers just beneath the lichen cover in lichen-dominated ecosystems of tundra are abundantly colonized by phylogenetically diverse assemblages of planctomycetes. Although the bacterial communities associated with lichens are commonly dominated by the Alphaproteobacteria, 16S rRNA gene sequences from the *Planctomycetes* have also been recovered from several lichen species by using high-throughput sequencing approach (Bates et al., 2011; Bjelland et al., 2011). These sequences, however, make only a minor proportion of all 16S rRNA gene reads obtained from lichen thalli suggesting that planctomycetes do not play a distinct functional role within lichen symbioses. Instead, planctomycetes appear to be involved in degradation of lichen-derived organic matter. Lichens are an important component of the vegetation cover in many ecosystems of tundra and degradation of lichen-derived litter is the basis of the microbial food chain in these ecosystems. The data obtained in our study are in agreement with the earlier observations that the organic layer serves as a hotspot of microbial abundance in tundra soils (Lee et al., 2013) and that members of the *Planctomycetes* are more abundant in the upper layer of a subarctic tundra soil (Kim et al., 2014).

The population number of planctomycetes in lichen-dominated tundra ecosystems was comparable to that reported earlier for boreal *Sphagnum* peat bogs, i.e., up to 10^7^ cells per gram of wet peat (Ivanova and Dedysh, 2012). The diversity of planctomycetes in these ecosystems, however, was somewhat different. Most 16S rRNA gene sequences representing this phylum in *Sphagnum*-derived peat commonly affiliate with the phylogenetic lineage defined by the genera *Singulisphaera* and *Isosphaera*, i.e., members of the family Isosphaeraceae (Serkebaeva et al., 2013; Moore et al., 2015). These bacteria were also present in lichen-dominated tundra ecosystems (**Figures 4** and **6**) but they did not comprise the dominant planctomycete group. As suggested by our cultivation-based studies, members of the Isosphaeraceae from tundra environments are better adapted for growth at low temperatures than those from boreal peatlands and, most likely, are represented by different species.

According to RDP classifier retrained with Silva 119 database used in our study, one of the most abundant planctomycete groups detected in lichen-dominated tundra ecosystems was the *Phycisphaera*-related soil group WD2101. This group was named after the environmental 16S rRNA gene sequence WD2101 (GenBank accession no. AJ292687) retrieved by Nogales et al. (2001) from an acidic polychlorinated biphenyl-polluted soil near Wittenberg, Germany. This cloned sequence revealed only a very distant relationship to known in 2001 members of the Planctomycetales and was therefore defined as a novel bacterial lineage, WPS-1. Members of this lineage have been detected by cultivation-independent approaches in a wide variety of soil environments. The first and so far the only characterized representative of this lineage, which is now defined as the class Phycisphaerae and the order Tepidisphaerales (and implemented as such in the recently released Silva 128), was described last year. This is the moderately thermophilic planctomycete from terrestrial hot springs, *Tepidisphaera mucosa* (Kovaleva et al., 2015). Similar to other described members of the Phycisphaerae, this planctomycete divides by binary fission and is capable of degrading various polysaccharides. *T. mucosa* grows between 20 and 56°C and in the pH range 4.5–8.5, with optimal growth at 47–50°C and pH 7.0–7.5.16S rRNA gene sequence similarity between *T. mucosa* and the corresponding gene fragments retrieved in our study is low (80.5–91.7%), suggesting that tundra-inhabiting members of this order belong to as-yet-undescribed family and may possess different temperature and pH adaptations defined by their habitat conditions. Our numerous attempts to isolate these bacteria by using the above described cultivation strategy and medium M31, as well as medium M1 supplemented with lichenan, xylan or xanthan gum as growth substrates, were unsuccessful. Obtaining isolates of these planctomycetes, therefore, represents a challenge for further studies.

The second most abundant group of 16S rRNA gene reads recovered in our study belonged to uncultivated members of the family Planctomycetaceae (**Figure 4**). As seen from phylogenetic tree in **Figure 6**, these sequences formed a large cluster phylogenetically related to the lineage defined by the genera *Gemmata*–*Zavarzinella*–*Telmatocola*. Close GenBank matches to these sequences also originated from various soil environments, including forest soils, agricultural fields, rice paddies, and prairies (**Table 2**; Supplementary Table S1). Apparently, these sequences represent a large and as-yet-uncultured sub-group of soil-inhabiting organisms within the Planctomycetales. Notably, only a very few reads retrieved from tundra soils affiliated with *Pirellula*-like planctomycetes. The latter are highly characteristic for marine environments and represent an important part of the complex microbial biofilm community of a wide range of macroalgae (Lage and Bondoso, 2014). These planctomycetes, however, are nearly absent from tundra soil environments.

In summary, our study identified lichen-dominated soils of tundra as a rich source of novel planctomycete diversity. Only 24–27% of 16S rRNA reads examined in our study could be assigned to the currently described genera of these bacteria. Two groups of as-yet-uncultivated soil planctomycetes revealed in tundra environments, i.e., a WD2101-like group within the Tepidisphaerales and a *Gemmata*-related group within the Planctomycetales, represent the most attractive objects for further isolation studies. The functional role of planctomycetes in lichen-dominated soils remains to be clarified. As suggested by substrate utilization tests with *Singulisphaera*-related isolates obtained in our study, planctomycetes are potentially able to participate in degradation of lichen debris. The role of secondary degraders, however, cannot be excluded since planctomycetes were identified as efficient degraders of exopolysaccharides produced by other soil bacteria (Wang et al., 2015). The specific position of planctomycetes in the microbial food chain in lichen-dominated ecosystems of tundra as well as the environmental factors that determine their activity *in situ* remain to be elucidated. It should also be noted that the sampling effort made in our study was limited by one time point only, which is clearly insufficient for creating a complete picture of planctomycete diversity and abundance in lichen-dominated ecosystems of tundra. Assessing the environmental variables that determine specific differences in diversity patterns of these bacteria in distinct ecosystems, such as wetlands and upland soils, also requires further studies with increased sampling effort, in order to account for the spatial heterogeneity in these habitats.

## Author Contributions

SD and AI designed the study, interpreted the results, and wrote the manuscript. IK performed isolation studies and cultivation experiments. ST and AM retrieved the sequence data set. AI analyzed the sequence data set. AM designed the *Planctomycetes*-specific probe.

## Conflict of Interest Statement

The authors declare that the research was conducted in the absence of any commercial or financial relationships that could be construed as a potential conflict of interest.
