# Supplementary material for: High Diversity of Planctomycetes in Soils of Two Lichen-Dominated Sub-Arctic Ecosystems of Northwestern Siberia
Source: Front Microbiol. 2016 Dec 22;7:2065. doi: 10.3389/fmicb.2016.02065 (PMC5177623; doi:10.3389/fmicb.2016.02065)
Supplement: Supplementary file 1 [file Table_1.PDF]

Table S1. Unique OTUs in a forested tundra soil (FT) and a shallow tundra peatland (PT).

OTU ID	Absolute abundance of OTUs		Taxonomy	Close GenBank match	Reported habitat	Similarity (%)
	PT	FT				
OTU37	0	605	Planctomycetaceae uncultured	JN002870	serpentinezed dunite, Norway	98
OTU38	0	438	Planctomycetaceae uncultured	GQ480057	activated sludge, China	94
OTU39	294	0	Phycisphaerae WD2101 soil group	EF516972	grassland soil, CA, USA	95
OTU40	0	521	Planctomycetaceae uncultured	KX753006	potato field soil, Czech Rep	93
OTU41	0	573	Planctomycetaceae uncultured	KX753006	potato field soil, Czech Rep	100
OTU42	0	272	Planctomycetaceae uncultured	KX753006	potato field soil, Czech Rep	95
OTU43	0	179	Planctomycetaceae uncultured	AB486049	rice paddy soil, Japan	97
OTU44	0	249	Planctomycetaceae uncultured	X64375	soil, Australia	94
OTU45	0	194	Planctomycetaceae uncultured	KX753070	potato field soil, Czech Rep	98
OTU46	0	111	Planctomycetaceae uncultured	GQ339148	Freshwater seep, Denmark	95
OTU47	0	250	Planctomycetaceae uncultured	X64375	soil, Australia	97
OTU48	0	108	Singulisphaera	HM362508	decomposting bagasse, Thailand	97
OTU49	0	106	Planctomycetaceae uncultured	EF516204	grassland soil, CA, USA	95
OTU50	0	122	Planctomycetaceae uncultured	EF018875	aspen rhizosphere, USA	93
OTU51	0	215	Planctomycetaceae uncultured	EU135181	grass prairie soil, USA	96
OTU52	0	126	Planctomycetaceae uncultured	EU044223	river valley soil, USA	99
OTU53	0	298	Singulisphaera	EF516307	grassland soil, CA, USA	98
OTU54	0	173	Planctomycetaceae uncultured	KM221442	hot spring, China	97
OTU55	0	286	Planctomycetaceae uncultured	KJ407477	forest soil, Taiwan	97
OTU56	0	151	Planctomycetaceae uncultured	EF018393	aspen rhizosphere, USA	96
OTU57	0	105	Planctomycetaceae uncultured	LN573921	ant refuse dumps, Panama	95
OTU58	0	235	Planctomycetaceae uncultured	LK025533	Peat soil, Germany	97
OTU59	0	131	Planctomycetaceae uncultured	X64375	soil, Australia	97

OTUs with the absolute abundance of >100 are shown.
