# Supplementary material for: High Diversity of Planctomycetes in Soils of Two Lichen-Dominated Sub-Arctic Ecosystems of Northwestern Siberia
Source: Front Microbiol. 2016 Dec 22;7:2065. doi: 10.3389/fmicb.2016.02065 (PMC5177623; doi:10.3389/fmicb.2016.02065)
Supplement: Supplementary file 2 [file Table_2.PDF]

Table S2. Statistical analysis of bacterial and archaeal sequence abundances shown in Fig. S2

Taxa	Significance	P value	t ratio	Q value
Archaea;Thaumarchaeota	No	0.95	0.06	0.88
Bacteria;Acidobacteria	**Yes**	**1E-15**	**11.02**	**1E-15**
Bacteria;Actinobacteria	No	0.03	2.22	0.12
Bacteria;Armatimonadetes	No	0.78	0.28	0.88
Bacteria;Bacteroidetes	No	0.88	0.15	0.88
*Bacteria;*Candidate division OD1	No	0.98	0.03	0.88
Bacteria;Chlamydiae	No	0.78	0.28	0.88
Bacteria;Chloroflexi	No	0.46	0.74	0.88
Bacteria;Cyanobacteria	No	0.90	0.12	0.88
Bacteria;Elusimicrobia	No	0.93	0.09	0.88
Bacteria;Firmicutes	No	0.69	0.40	0.88
Bacteria;Gemmatimonadetes	No	0.90	0.12	0.88
Bacteria;Planctomycetes	**Yes**	**2.1E-06**	**5.16**	**1.7E-05**
Bacteria;Proteobacteria	No	0.39	0.86	0.88
Bacteria; Candidate division TM6	No	0.93	0.09	0.88
Bacteria;Verrucomicrobia	No	0.01	2.56	0.07
Bacteria; Cand. division WD272	No	0.74	0.34	0.88
Bacteria;Other	No	0.83	0.22	0.88

OTUs with the absolute abundance of >100 are shown.

Statistical evaluations were made applying multiple t tests between datasets from a forested tundra soil and a shallow peatland (both datasets in three replicates). Significant differences are indicated with bold.
