# Supplementary material for: High Diversity of Planctomycetes in Soils of Two Lichen-Dominated Sub-Arctic Ecosystems of Northwestern Siberia
Source: Front Microbiol. 2016 Dec 22;7:2065. doi: 10.3389/fmicb.2016.02065 (PMC5177623; doi:10.3389/fmicb.2016.02065)
Supplement: Supplementary file 3 [file Table_3.PDF]

Table S3. Statistical analysis of *Planctomycete* sequence abundances shown in Figure 4

Taxa	Significance	P value	t ratio	Q value
BD7-11 uncultured group	No	0.98	0.02	0.78
vadinHA49	No	0.99	0.01	0.78
***Phycisphaerae* WD2101 soil group**	**Yes**	**5.6E-10**	**8.38**	**1.9E-09**
Phycisphaeraceae Phycisphaera	No	0.96	0.05	0.78
Gemmata	No	0.59	0.54	0.78
Planctopirus/Rubinisphaera	No	0.95	0.07	0.78
Isosphaera	No	0.86	0.18	0.78
Singulisphaera	No	0.95	0.07	0.78
***Planctomycetaceae* uncultured**	**Yes**	**5.6E-11**	**9.19**	**3.9E-10**

Statistical evaluations were made applying multiple t tests between datasets from a forested tundra soil and a shallow peatland (both datasets in three replicates). Significant differences are indicated with bold.
