# Supplementary material for: High Diversity of Planctomycetes in Soils of Two Lichen-Dominated Sub-Arctic Ecosystems of Northwestern Siberia
Source: Front Microbiol. 2016 Dec 22;7:2065. doi: 10.3389/fmicb.2016.02065 (PMC5177623; doi:10.3389/fmicb.2016.02065)
Supplement: Supplementary file 4 [file Image_1.PDF]

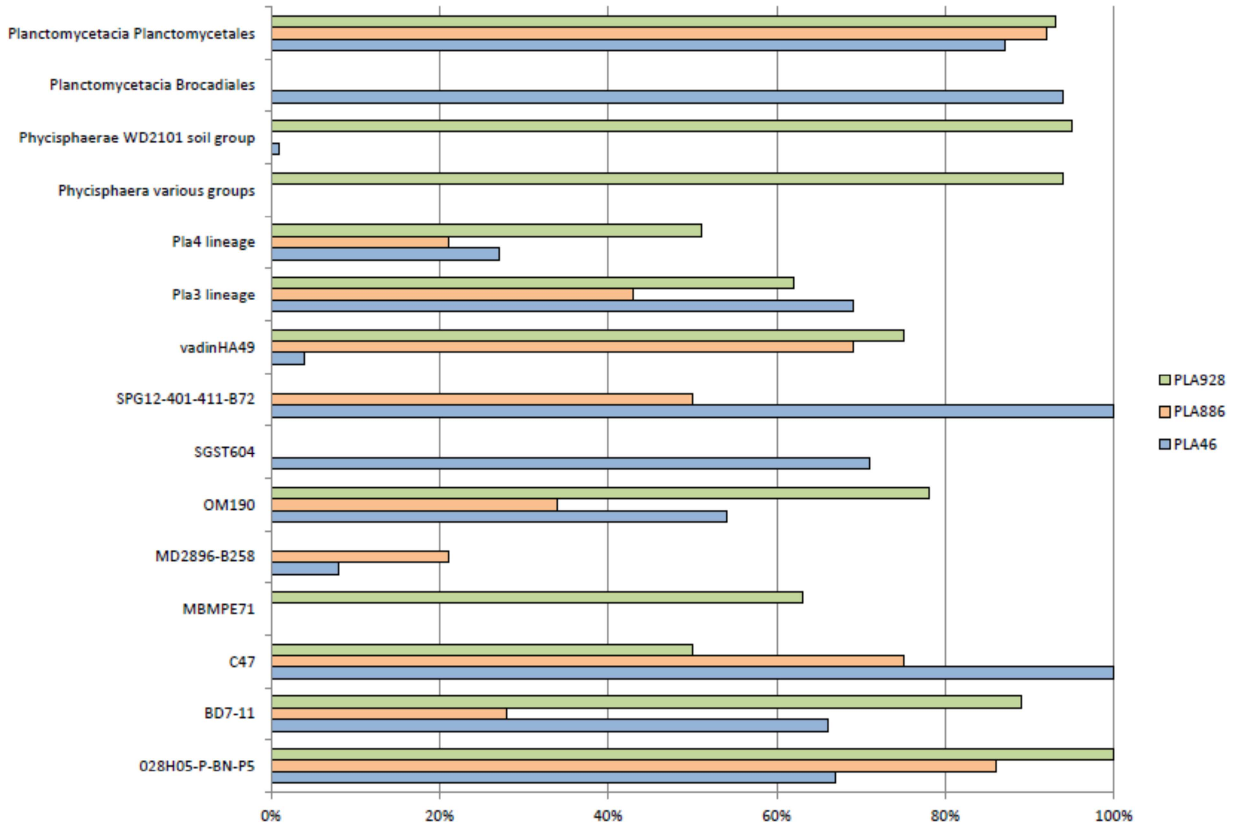

Supplementary Figure S1. Target specificity of the *Planctomycetes*-specific probes PLA46 (blue), PLA886 (orange) and PLA929 (green). The probe match analysis was performed using SILVA 119 database.
