# Supplementary material for: High Diversity of Planctomycetes in Soils of Two Lichen-Dominated Sub-Arctic Ecosystems of Northwestern Siberia
Source: Front Microbiol. 2016 Dec 22;7:2065. doi: 10.3389/fmicb.2016.02065 (PMC5177623; doi:10.3389/fmicb.2016.02065)
Supplement: Supplementary file 5 [file Image_2.PDF]

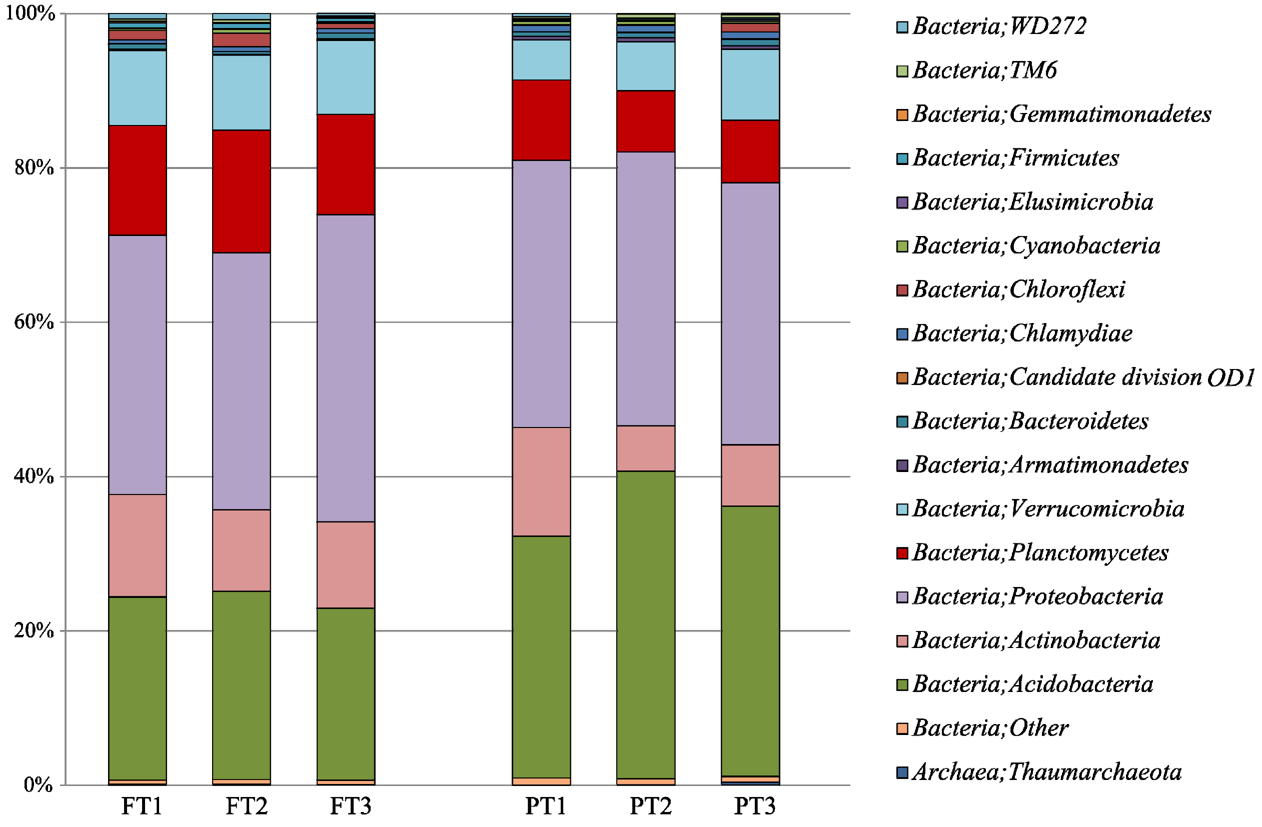

Supplementary Figure S2. Microbial community composition in 3 sites of the forested tundra (FT) and 3 peatland sites (PT) based on Illumina paired-end sequencing of 16S rRNA genes.
