# Supplementary material for: High Diversity of Planctomycetes in Soils of Two Lichen-Dominated Sub-Arctic Ecosystems of Northwestern Siberia
Source: Front Microbiol. 2016 Dec 22;7:2065. doi: 10.3389/fmicb.2016.02065 (PMC5177623; doi:10.3389/fmicb.2016.02065)
Supplement: Supplementary file 6 [file Image_3.PDF]

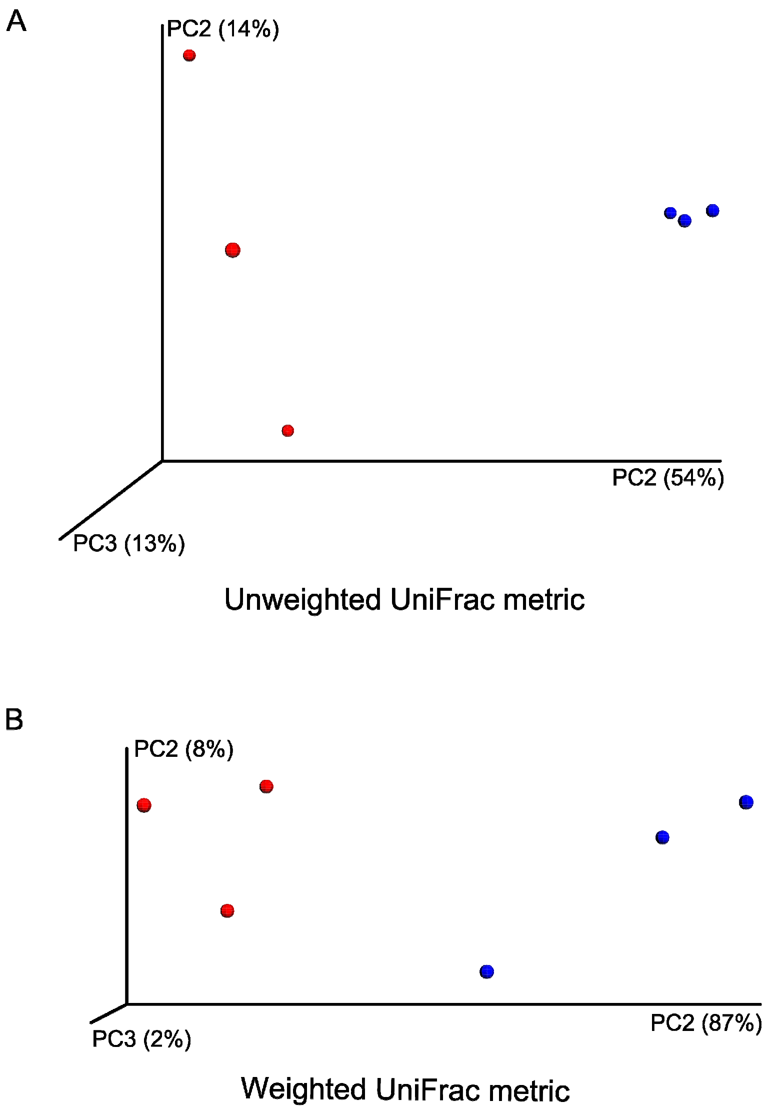

Supplementary Figure S3. Principal coordinates analysis of (A) Unweighted and (B) Weighted UniFrac values between forested tundra soil (blue) and a peatland (red) sequencing datasets with the axes scaled by the percentage of the variance that they contain.
